# Fipronil and ivermectin treatment of cattle reduced the survival and ovarian development of field-collected *Anopheles albimanus* in a pilot trial conducted in northern Belize

**DOI:** 10.1186/s12936-019-2932-6

**Published:** 2019-08-29

**Authors:** Staci M. Dreyer, Donovan Leiva, Marla Magaña, Marie Pott, Jonathan Kay, Alvaro Cruz, Nicole L. Achee, John P. Grieco, Jefferson A. Vaughan

**Affiliations:** 1 Biology Department, University of North Dakota, Grand Forks, ND 58202 USA; 2 Belize Vector and Ecology Center, Orange Walk Town, Belize; 3 University of Notre Dame, South Bend, IN 46556 USA

**Keywords:** Malaria, *Anopheles albimanus*, Belize, Ivermectin, Fipronil, Cattle

## Abstract

**Background:**

Most malaria vector control programmes rely on indoor residual spraying of insecticides and insecticide-treated bed nets. This is effective against vector species that feed indoors at night and rest inside the house afterwards. In Central America, malaria vectors have different behaviours and are typically exophagic (i.e., bite outdoors), exophilic (i.e., remain outdoors after feeding), and zoophagic (i.e., as likely to feed on non-humans as humans). Thus, malaria elimination in Central America may require additional tactics. This pilot study investigated whether commercially-available products used to treat livestock for ticks could also be used to kill and/or sterilize zoophagic malaria vectors that feed on treated cattle in Belize.

**Methods:**

Cattle were treated with either a pour-on formulation of 1% fipronil (3 heifers) or injection of 1% ivemectin (1 heifer). Control heifers (n = 2) were left untreated. Field-collected *Anopheles albimanus* contained in screen-top cages were strapped onto cattle at 2, 5, 7, and 14 days after treatment. Mosquito mortality was monitored once a day for 4 successive days. Surviving mosquitoes were dissected to assess blood meal digestion and ovarian development.

**Results:**

A total of 1078 female *An. albimanus* mosquitoes were fed and monitored for mortality. Both fipronil and ivermectin significantly reduced survivorship of *An. albimanus* for up to 7 days after treatment. By 14 days, efficacy had declined. The ivermectin treatment completely lost its effectiveness and even though the fipronil-treated heifers were still killing significantly more mosquitoes than the untreated heifers, the amount of mosquito killing had diminished greatly. Both treatments significantly reduced ovary development in mosquitoes fed on treated cattle for the duration of the 2-week trial.

**Conclusions:**

Treatment of cattle in northern Belize with topical fipronil and injectable ivermectin had significant lethal and sublethal effects on wild *An. albimanus* females. These results suggest that efforts towards eliminating residual transmission of malaria by zoophagic vectors in Central America may benefit by the judicious, targeted treatment of livestock with mosquitocidal compounds, such as fipronil or ivermectin.

## Background

Only a small proportion of the worldwide malaria burden is attributed to Central and South America [1]. Currently, several countries in this region are in the process of elimination certification, while others are projected to eliminate malaria by 2020, including Belize [1]. To eliminate the residual transmission of malaria, vector control tactics must consider the diversity of mosquito feeding behaviours that occur within the Americas. Many vectors in this region tend to be exophagic (feed outdoors), exophilic (rest outdoors), and zoophagic (feed more preferentially on animals than humans) [2]. Such behaviours render the standard vector control methods of indoor residual spraying and insecticide-impregnated bed nets less effective. Endectocide use in livestock has been suggested as a novel control method that can effectively target vectors with these behavioural traits.

The avermectin class of endectocides such as ivermectin and eprinomectin, have a long history of use in humans and livestock as ‘de-worming’ agents against parasitic nematodes. Avermectins bind selectively to glutamate-gated chloride ion channels in the neurons of nematodes and arthropods. Binding leads to an influx chloride ions, causing hyperpolarization at the synapse and neuromuscular junction. Paralysis and death of the parasite ensues. Mosquito ingestion of ivermectin in particular has been demonstrated to reduce post-feeding survival, egg development and fecundity, blood meal digestion, and re-feeding behaviour in a number of Old World [3–15] and New World [16–19] *Anopheles* species. Similarly, treating cattle with eprinomectin has been shown to reduce the survival of *Anopheles arabiensis* in Kenya [6]. The phenylpyrazole compound, fipronil is a broad-spectrum insecticide/acaricide, commonly used for flea and tick control in companion animals. Fipronil blocks GABA-gated ion channels in the central nervous system of arthropods, thereby blocking inhibitory neuron transmission and resulting in hyper-excitability and death of susceptible arthropods. Fipronil is not registered for use for livestock within the USA, but several brands of fipronil products are registered for use in livestock against cattle ticks and biting flies in certain Latin American countries. Fipronil, administered to cattle either orally or topically has been shown to significantly reduce survival of phlebotomine sand flies [20] and tsetse [21]. Importantly, cattle ingesting technical grade fipronil at a dose of 1.5 mg/kg BW have been shown to significantly reduce the post-feeding survival of the zoophagic African vector, *An. arabiensis*, for up to 21 days after cattle dosing [6].

The goal of this pilot study was to evaluate survival of wild-caught *Anopheles albimanus*, a dominant Central American malaria vector, after feeding on fipronil- and ivermectin-treated cattle in Orange Walk District in North-Central Belize. *Anopheles albimanus* displays exophagic, exophilic and zoophagic behaviours [2] and thus is an appropriate species in which to test this method of vector control. How long the treatments retained their insecticidal activity over time (i.e., residual activity), as well as any potential sub-lethal effects that the treatments might have on mosquito blood meal digestion and oogenesis were also monitored.

## Methods

### Mosquitoes

Host-seeking mosquitoes were collected at night by human landing catches in San Roman Rio Hondo, Orange Walk District, Belize. Mosquitoes were transported to the Belize Vector and Ecology Center (BVEC) laboratory in Orange Walk Town, Belize. *Anopheles albimanus* mosquitoes were distinguished from other anopheline species routinely collected in San Roman (e.g., *Anopheles punctimacula, Anopheles vestitipennis*) based on the characteristic banding pattern on the hind tarsi of *An. albimanus* [22]. Mosquitoes were maintained at 26 °C with access to 8% honey solution ad libitum. Mosquito collections were conducted for two consecutive nights prior to each cattle feed. To estimate potential changes in the age structure of the mosquitoes used during the experiment, all mosquitoes that had not blood-fed at the conclusion of each feeding trial were dissected and scored as either nulliparous or parous, based on the presence (= nulliparous) or absence (= parous) of tracheolar coiling or skeins on the surface of the ovaries [23–25]. Parity rate was expressed as the percentage of parous mosquitoes.

### Cattle treatment and mosquito feeds

The experiment was conducted on a cattle ranch near the village of San Felipe, Orange Walk District, Belize, with the informed consent of the ranch owner. Six healthy heifers, Brahma (*Bos taurus indicus*)-Brown Swiss (*Bos taurus*) hybrid mix ranging from 315 to 430 kg, were randomly selected and rounded up by the owner and his wrangler on horseback from a herd grazing in a nearby field. The herd had not received insecticidal or acaricidal treatment for at least 6 months prior to initiating the experiment. Heifers were driven into a holding corral that contained a cattle alley and squeeze chute. Each heifer had a numbered identifying ear tag and was randomly allocated to either one of two treatment groups or the control group. Three heifers received Ectonil^®^ Pour-on (1% fipronil) (Agrovetmarket, Lima PERU) following the instructions on the label for control of ticks. The product was dispensed along the dorsal midline from the neck to the base of the tail at a rate of 5 ml per 50 kg body weight. One heifer received Labimectin^®^ (1% ivermectin) (LabiPharma, Guatemala City, GUATEMALA) following the instruction on the label for control of intestinal roundworms. The product was administered as an intramuscular injection at a dose of 1 ml per 50 kg body weight. Two heifers remained untreated and served as control animals. Because the fipronil was applied dermally, the three fipronil-treated heifers were separated from the other heifers for 48 h after treatment to prevent the possibility of cross contamination due to normal huddling and herding activity of cattle. Afterwards, the heifers were pastured together.

Prior to treatment, a pre-treatment mosquito feeding was conducted on each heifer in order to (1) establish baseline information on mosquito feeding rates and post-feeding survival, and (2) optimize procedures for handling the cattle and conducting controlled mosquito feeds. Two styles of polypropylene containers were tested for their suitability as feeding chambers; modified flat rectangular food storage containers and modified beverage cups. Both had screened lids through which mosquitoes could feed and screened windows cut into the sides to reduce build-up of condensation while attached to the heifer. Mosquito feeding rates and survival were better in the modified beverage cups; therefore, cups were used for the remainder of the study. Feeding cups were re-used between trials. However, to avoid potential residue contamination, filter paper inserts placed on the bottom of the cups and screen mesh coverings were replenished between each use. In addition, cups were cleaned with isopropyl alcohol then exposed to sunlight for one or 2 days between uses to promote photodegradation of any potential insecticidal residues.

Experimental mosquito feedings were conducted at 2, 5, 7, and 14 days after cattle treatment. The day before each feeding trial, mosquitoes were placed into feeding cups (15 to 40 per cup). Feeding cups were transported to the ranch by automobile (ca. 40 min) in an uncovered cooler to reduce formation of condensation in the cups. Feedings were conducted in the late afternoon (ca. 1530 to 1730 h, local time). Cattle were herded into a corral that had at one side, an alley that led into a metal squeeze chute where cattle could be individually restrained during the mosquito feeding procedure. Once a heifer was in the squeeze chute, two areas near the midline were shaved using a small battery-operated livestock clipper. Two cups were selected at random and secured to the animal, one on each side, by encircling the animal’s midsection 2–4 times with plastic wrap. Mosquitoes were allowed to feed for 15 min, then the plastic was cut, and the cups were removed and transported back to the BVEC. Unfed and partially-fed mosquitoes were removed with a glass aspirator, verified visually under low magnification, and then expelled into another, larger cage for dissection and parity determinations the following morning (see above). Fully engorged mosquitoes were maintained indoors in a temperature-controlled room at 24 °C and access to cotton pledgets soaked in 8% honey solution.

### Mosquito mortality, digestion and ovarian development

Mosquito mortality was assessed by counting and removing dead mosquitoes from each feeding cup every day. At the end of 4 days, surviving mosquitoes were counted and dissected to assess blood meal digestion and ovarian development. Blood meal digestion was scored as either negative (no traces of blood in the midgut) or positive (blood present in the midgut). Ovarian development was scored as fully gravid (ovaries with fully developed ovarioles), half gravid (ovaries enlarged but ovarioles not fully developed), or not gravid (small ovaries with no ovariole development) [23–25].

### Data analyses

Mosquito survivorship was analyzed with a Kaplan–Meier survival analysis and Logrank test (GraphPad Software, La Jolla, CA, USA). Sub-lethal effects on ovarian development and blood meal digestion were analysed using generalized linear mixed effects model (GLMM) in R (R package “lme4”) [26, 27]. The GLMM analysis was chosen because it provided greater flexibility when examining the categorical and continuous explanatory variables, fit well for binomial response variable, and accounted for random effects (i.e., heifer). After running full models, sub models were constructed to identify the best overall model that explained the data, based on AIC and null deviance. Odds ratios with their respective confidence intervals were calculated in R, using the standard error of the models. A 0.05 level of significance was used throughout.

## Results

**Fig. 1:**
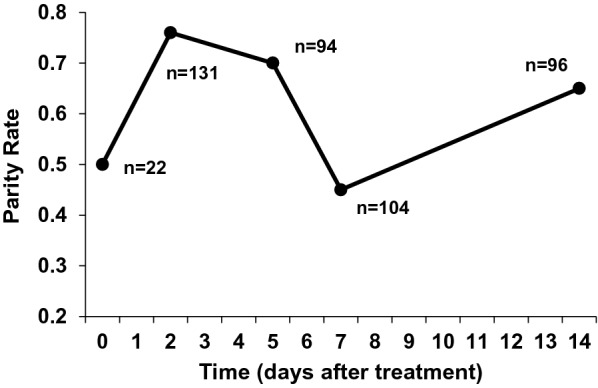
Parity status of wild-caught *Anopheles albimanus* mosquitoes used in cattle feeding. Orange Walk District, Belize. 28 June to 12 July 2018

To estimate the age structure of the mosquitoes used in this trial, the ovaries of unfed mosquitoes were excised and 373 were successfully scored for parity. The overall parity rate was 62% (range 45% to 76%). The age structure of mosquitoes used during this study fluctuated over the 2-week course of the study. Mosquitoes used at day 7 were physiologically younger (i.e., lower parity rate) than were mosquitoes used at days 2, 5, and 14 after treatment (Fig. 1). A substantial proportion of *An. albimanus* (26% of 382 examined) had an unknown species of larval ectoparasitic water mite attached to their thoraces and abdomen (Acari: Hydarchinida). Infestations were generally light (geometric mean intensity = 1.7 mites per infested mosquito).

A total of 1783 wild-caught *An. albimanus* were exposed to cattle of which 1078 (60%) took a blood meal. There was no difference between the feeding successes of *An. albimanus* on fipronil-treated heifers versus those on untreated control heifers (Table 1) throughout the trial. However at days 2 and 5 after treatment, the feeding success of *An. albimanus* on the ivermectin-treated heifer was significantly lower than those on both the untreated control heifers and fipronil-treated heifers. The heifer treated with ivermectin did not seem to be inherently repellent to mosquitoes because prior to treatment the mosquito feeding rate on this animal (37.5%, N = 48) did not differ statistically from mosquito feeding rates on the other five heifers prior to treatment (47.6%, N = 252) (χ^2^ = 1.66, p = 0.20). Mosquito repellency of the ivermectin-injected heifer dissipated within a week (Table 1).

**Table 1:** Proportion (± 95% confidence interval) of wild-caught *Anopheles albimanus* that ingested blood when exposed to treated and untreated heifers (Belize, 2018)

Treatment	Number of heifers	Pre-treatment	Day after treatment			
			Day 2	Day 5	Day 7	Day 14
Control	2	43 ± 9% N = 108/n_1_ = 48, n_2_ = 60	54 ± 10% N = 100/n_1_ = 50, n_2_ = 50	62 ± 9% N = 120/n_1_ = 60, n_2_ = 60	68 ± 11% N = 71/n_1_ = 36, n_2_ = 35	79 ± 6% N = 192/n_1_ = 96, n_2_ = 96
Fipronil	3	51 ± 8% N = 144/n_1_ = 48, n_2_ = 48, n_3_ = 48	47 ± 8% N = 150/n_1_ = 50, n_2_ = 50, n_3_ = 50	65 ± 7% N = 176/n_1_ = 58, n_2_ = 58, n_3_ = 60	71 ± 9% N = 102/n_1_ = 34, n_2_ = 34, n_3_ = 34	74 ± 5% N = 288/n_1_ = 96, n_2_ = 96, n_3_ = 96
Ivermectin	1	37% N = 48	24% N = 50	40% N = 57	51% N = 33	85% N = 96
Statistical comparisons	Control vs. fipronil	χ^2^ = 1.91 p = 0.17	χ^2^ = 1.07 p = 0.30	χ^2^ = 0.25 p = 0.62	χ^2^ = 0.18 p = 0.67	χ^2^ = 1.38 p = 0.24
	Control vs ivermectin	χ^2^ = 0.36 p = 0.60	χ^2^ = 12.2 p = 0.0005	χ^2^ = 7.7 p = 0.006	χ^2^ = 2.49 p = 0.11	χ^2^ = 1.90 p = 0.17

*N* total number of mosquitoes exposed to a treatment group; *n* number of mosquitoes exposed per heifer within a treatment group

**Fig. 2:**
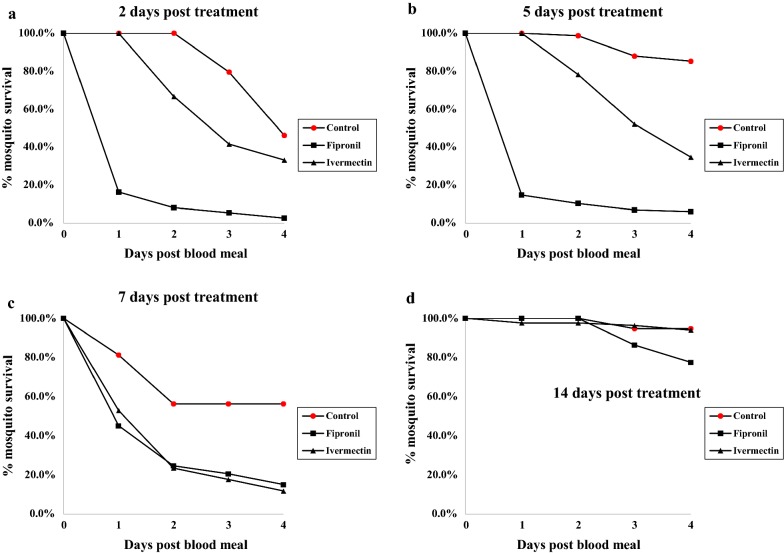
Residual mosquitocidal activity of cattle treated with either fipronil (topical application) or ivermectin (intramuscular injection) via monitoring the daily survival of *Anopheles albimanus* mosquitoes for 4 days after blood feeding on treated *versus* untreated cattle. **a** Mosquitoes fed 2 days after cattle treatment, **b** mosquitoes fed 5 days after cattle treatment, **c** mosquitoes fed 7 days after cattle treatment, **d** mosquitoes fed 14 days after cattle treatment

Throughout the duration of the trial, the median survival of mosquitoes feeding on fipronil-treated heifers was significantly less (p < 0.05) than the median survival of mosquitoes fed on untreated heifers (Table 2; Fig. 2). Although overall mosquitocidal efficacy of fipronil treatments deteriorated by day 14 (see Fig. 2), the 4-day survival curve of mosquitoes fed on fipronil-treated heifers remained significantly different than the survival curve of mosquitoes fed on untreated heifers (Table 2). At 2, 5, and 7 days after cattle treatment, the median survival of mosquitoes fed on the ivermectin-treated heifer was significantly less (p < 0.05) than the median survival of mosquitoes fed on untreated heifers (Table 2; Fig. 2). Efficacy of the ivermectin treatment dissipated during the second week and at day 14, the median survival of mosquitoes fed on the ivermectin-treated heifer did not differ significantly from the median survival of mosquitoes fed on untreated heifers (Table 2).

**Table 2:** Median survival in days of wild-caught *Anopheles albimanus* fed on treated and untreated heifers (Belize 2018)

	Pre-treat	Day 2	Day 5	Day 7	Day 14
Untreated heifers (n = 2)					
Mosquito survival (days)	> 4* (n = 46)	4 (n = 54)	> 4* (n = 75)	> 4* (n = 48)	> 4* (n = 151)
Fipronil-treated heifers (n = 3)					
Mosquito survival (days)	> 4* (n = 74)	1 (n = 73)	1 (n = 115)	1 (n = 72)	> 4* (n = 213)
Statistical significance**	–	χ^2^ = 96.8; p < 0.0001	χ^2^ = 150.7; p < 0.0001	χ^2^ = 23.7; p < 0.0001	χ^2^ = 19.6; p < 0.0001
Ivermectin-treated heifer (n = 1)					
Mosquito survival (days)	> 4 (n = 18)	3 (n = 12)	4 (n = 23)	2 (n = 17)	> 4* (n = 82)
Statistical significance**	–	χ^2^ = 4.2; p = 0.04	χ^2^ = 27.1; p < 0.0001	χ^2^ = 11.7; p = 0.0006	χ^2^ = 0.08; p = 0.78

* Median mosquito survival exceeded the 4 day observation period; n = total number of engorged mosquitoes

** The χ^2^ and p-values compare survival curves of the treated groups with that of the corresponding untreated control group, as determined by Logrank tests

Mosquito death after feeding on fipronil-treated heifers was rapid (Fig. 2). Many fipronil-treated mosquitoes became moribund within hours after feeding and the majority of mosquito mortality occurred within 24 h (median survival times = 1 day). In contrast, mosquitoes fed on the ivermectin-treated heifer took several days to die (median survival times = 3 to 4 days) (Table 2).

Overall, 85% of mosquitoes that fed on untreated heifers contained fully gravid ovaries at 4 days after blood feeding (N = 228; range 78 to 91%) (Table 3). Mosquitoes fed on fipronil-treated heifers were significantly less likely to have fully developed ovaries compared to mosquitoes fed on untreated heifers (Table 4, p < 0.0001, OR: 0.0314) and when averaged over the 14-day period, only 22% (N = 183; range 0 to 25%) of the mosquitoes fed on fipronil-treated heifers contained fully developed ovaries (Table 3). Likewise, mosquitoes fed on the ivermectin-treated heifer were less likely to have fully developed ovaries compared to mosquitoes fed on untreated heifers (Table 4, p = 0.0001, OR: 0.169) and when averaged over the 14-day period, only 58% (N = 89; range 50 to 60%) of the mosquitoes fed on the ivermectin-treated heifer contained fully developed ovaries (Table 3). The influence of ‘day after treatment’ on mosquito gravidity was borderline significant (Table 4, p = 0.07, OR: 1.062).

**Table 3:** Ovarian development and blood digestion in *Anopheles albimanus* surviving 4 days after feeding on cattle

	Pre-treat	Day 2	Day 5	Day 7	Day 14
Untreated heifers (n = 2)					
Fully gravid	78 ± 14%	80 ± 16%	91 ± 7%	78 ± 16%	86 ± 8%
Complete blood meal digestion	91 ± 10%	100%	91 ± 7%	93 ± 10%	99 ± 7%
No. mosquitoes dissected	N = 32/n_1_ = 27, n_2_ = 5	N = 25/n_1_ = 12, n_2_ = 13	N = 64/n_1_ = 29, n_2_ = 35	N = 27/n_1_ = 11, n_2_ = 16	N = 80/n_1_ = 40, n_2_ = 40
Fipronil-treated heifers (n = 3)					
Fully gravid	83 ± 15%	^a^ N = 2/n_1_ = 2, n_2_ = 0, n_3_ = 0	0%	0%	25 ± 6%
Complete blood meal digestion	96 ± 8%		100%	100%	100%
No. mosquitoes dissected	N = 23/n_1_ = 7, n_2_ = 7, n_3_ = 9		N = 7/n_1_ = 0, n_2_ = 4, n_3_ = 3	N = 11/n_1_ = 1, n_2_ = 3, n_3_ = 7	N = 165/n_1_ = 47, n_2_ = 53, n_3_ = 65
Ivermectin-treated heifer (n = 1)					
Fully gravid	50%	50%	50%	^a^ N = 2	60%
Complete blood meal digestion	75%	75%	63%		90%
No. mosquitoes dissected	N = 4	N = 4	N = 8		N = 77

^a^Too few (N = 2) mosquitoes survived engorgement to make meaningful estimates. N = total number of surviving mosquitoes dissected within a treatment group; n = number of surviving mosquitoes dissected per heifer within a treatment group. Complete blood meal digestion was defined as the percentage of mosquitoes without visible evidence of blood in their midguts when excised and examined microscopically 4 days after engorgement. Fully gravid was defined as the percentage of mosquitoes with fully developed Stage V ovaries 4 days after engorgement

**Table 4:** Best-fit generalized linear mixed effects model (binomial) of variables and interactions that influenced ovary development

Fixed effects	Groups	Estimate	SE	*p* value	Odds ratio (95% CI)
Intercept		1.315	0.369	< 0.0004	3.724 (3.000, 4.448)
Treatment	Control (n = 196)	–	–	–	–
	Fipronil (n = 185)	− 3.379	0.407	< 0.0001	0.0314 (− 0.764, 0.832)
	Ivermectin (n = 91)	− 1.776	0.455	0.0001	0.169 (− 0.722, 1.061)
Day after treatment		0.060	0.033	0.07	1.062 (0.998, 1.126)

Model: FullyGravid ~ Treatment + DayAfterTreat + (1|CowID), where the fixed effects were ‘Treatment’ (n = total number of mosquitoes dissected per treatment group) and ‘Day After Treatment’. The random effect was ‘CowID’ (i.e., the six individual heifers. Standard deviation = 0.250)

Most (95%) of the 228 mosquitoes that fed on untreated heifers digested their blood meals completely by 4 days and retained no trace of blood residue within the midgut (Table 3). Similarly, all of 183 mosquitoes fed on fipronil-treated heifers completely digested their blood meals and fipronil had no effect on blood meal digestion (Table 5, p = 0.147, OR: 3.781). In contrast, ivermectin had a significant inhibitory effect on blood meal digestion (Table 5, p = 0.0019, OR: 0.201) and the proportion of fully digested blood meals in mosquitoes fed on the ivermectin-treated heifer at 2 and 7 days after treatment was 67% (N = 12) which was significantly less than in mosquitoes fed on either the control (χ^2^ = 10.3, p = 0.0013) or the fipronil-treated (χ^2^ = 36.2, p < 0.0001) heifers (Table 3). The influence of ‘day after treatment’ on mosquito digestion was significant (Table 5, p = 0.0318, OR: 1.121), indicating that as time passed, more mosquitoes were able to fully digest their blood meals.

**Table 5:** Best-fit generalized mixed effects model (binomial) of variables that influenced blood digestion

Fixed effects	Groups	Estimate	SE	p-value	Odds ratio (95% CI)
Intercept		2.181	0.486	< 0.0001	8.852 (7.900, 9.804)
Treatment	Control (n = 196)	–	–	–	–
	Fipronil (n = 185)	1.595	1.100	0.147	3.781 (2.772, 7.085)
	Ivermectin (n = 91)	− 1.661	0.534	0.0019	0.201 (− 0.856, 1.236)
Day after treatment		0.114	0.053	0.0318	1.121 (1.017, 1.225)

Model: BMneg ~ Treatment + DayAfterTreatment + (1|CowID) where the fixed effects were ‘Treatment’ (n = total number of mosquitoes dissected per treatment group) and ‘Day After Treatment’. The random effect was ‘CowID’ (i.e., the six individual heifers)

## Discussion

In a pilot trial conducted in northern Belize, treatment of heifers with a single dose of two commercially-available livestock parasiticides—Ectonil^®^ (1% fipronil pour-on formulation) and Labimectin^®^ (1% ivermectin injectable formulation)—each yielded significantly higher post-feeding mortality in field-collected *A albimanus* mosquitoes than did untreated control heifers. Of the two products, Ectonil^®^ was more effective, longer-lasting and produced significant, albeit declining, mosquito mortality for up to 2 weeks after cattle treatment. Efficacy of the Labimectin^®^ treatment lasted 1 week. In addition to lethal effects of the treatments, mosquitoes that survived feeding on treated heifers exhibited reduced ovarian development. The sterilizing effect lasted for the duration of the 2-week trial period but was much more pronounced in mosquitoes fed on fipronil-treated heifers.

Efficacy of ivermectin against field-collected *An. albimanus* was unexpected. Earlier laboratory studies demonstrated that ingestion of ivermectin-treated blood at concentrations normally found in the serum of treated cattle (i.e., 30–46 ng/ml) had no effect on *An. albimanus* mortality or ovarian development [28]. However, the strain of *An. albimanus* used in those laboratory studies (STECL strain) has been in continuous colony for many decades and may have been subject to intense inbreeding that somehow led to an ivermectin-resistant strain. One important factor found to contribute to ivermectin resistance in the STECL strain of *An. albimanus* was the poor absorption of ingested ivermectin across the gut [28]. There may be other mechanisms of resistance as well. Because metabolic resistance to both ivermectin and permethrin insecticide are mediated by common pathways—e.g., the cytochrome p450 system—a standardized CDC bottle bioassays on the STECL strain using permethrin was conducted to determine if this strain displayed metabolic resistance to permethrin. The STECL strain of *An. albimanus* was fully susceptible to permethrin (Additional file 1: Table S1). Thus, detoxification via the cytochrome p450 system does not appear to be an important mechanism of ivermectin resistance in the STECL strain of *An. albimanus*. Nevertheless, the marked difference in susceptibilities between a long-colonized strain of *An. albimanus* versus a wild population of field-collected *An. albimanus* mosquitoes highlights two important concepts. First, the large difference in susceptibilities emphasizes the importance of testing insecticides against genetically diverse populations of insects. On a more precautionary note, the susceptibility difference serves as a reminder that this *Anopheles* species (and probably others as well) has the capacity to develop resistance to ivermectin.

Both fipronil and ivermectin disrupt the inhibitory nervous system of insects but act in opposite ways. Fipronil blocks inhibitory neurotransmission, resulting in unregulated excitatory neurotransmission, hyper-excitability, and death. Conversely, ivermectin enhances inhibitory neurotransmission, resulting in paralysis and death. Not surprisingly, the physiological effects of fipronil and ivermectin on *An. albimanus* differed. Mosquito ingestion of fipronil produced rapid ‘knock-down’ and most of the mosquito mortality occurred within 24 h. Mortality in mosquitoes ingesting ivermectin was protracted and occurred over several days. Both compounds inhibited ovarian development, but only ivermectin inhibited blood meal digestion. There was a substantial degree of concordance between blood feeding and ovarian development in mosquitoes fed on untreated heifers, with 95% of 241 engorged mosquitoes fully digesting their blood meals and 85% subsequently developing gravid ovaries. In contrast, ingestion of fipronil resulted in gonotrophic discordance, with 100% of 183 engorged mosquitoes fully digesting their blood meals but only 22% subsequently developing gravid ovaries. The effect that this ‘fipronil-mediated gonotrophic discordance’ has on the frequency of re-feeding by surviving *An. albimanus* was not examined. However, if fipronil-mediated gonotrophic discordance were to enhance re-feeding in the survivors, then the outcome could either be desirable (i.e., if re-feeding occurred on treated cattle) or undesirable (i.e., if re-feeding occurred on humans). At this point, it is premature to speculate whether or not fipronil-mediated gonotrophic discordance would change the behaviour of a normally zoophagic population to one of anthropophagy.

Treatment of livestock with various formulations of fipronil and avermectin-based compounds such as ivermectin and eprinomectin, have been shown to significantly reduce the survival and fecundity of the zoophagic vectors, *An. arabiensis* in Africa [4, 6, 10], and *Anopheles culicifacies* and *Anopheles stephensi* in Pakistan [5]. This study extends those findings and demonstrates that treatment of cattle with commercial livestock products may also be employed in the Americas to control zoophagic vectors. The residual activities of the products used in our trial were relatively short-lived (≤ 2 weeks). Product formulation can influence the longevity of a compound’s residual effects. For example, recent studies describe the development and field-testing of a slow-release silicone-based ivermectin implant for livestock that extended the mosquitocidal activity of ivermectin out to 6 months [4, 29]. Advances in product formulation (e.g., slow-release implants) to extend a compound’s residual activity, identification of multiple mosquitocidal compounds with different modes of action (e.g., avermectins versus fipronil), and strategic development of staggered treatment regimens can all contribute to optimizing the “mosquitocidal livestock” strategy against zoophagic malaria vectors. As a practical matter, making cattle “poisonous” to zoophagic *Anopheles* vectors as part of a regional malaria control programme will be more acceptable to ranchers and, therefore, more likely to be integrated into their routine livestock management if (1) the compounds and formulations employed have regulatory approval by local agricultural health authorities, and (2) if the effort to treat cattle confers a tangible benefit to the livestock producer (e.g., tick control).

## Conclusions

This pilot study suggests that the treatment of cattle with commercially available livestock drugs can augment malaria elimination efforts in Central America. Further field studies employing larger sample sizes, pharmacokinetic observations, and a wider diversity of vector species, livestock species and drugs will be needed to fully assess the effectiveness of this approach in helping to extinguish residual malaria transmission in the Americas.

## Supplementary information

**Additional file 1.** Additional information on comparative toxicities of ivermectin versus permethrin for the STECL laboratory strain of *Anopheles albimanus.*

## Data Availability

The data analysed during this study are available on request from the corresponding author.

## Abbreviation

BVEC: Belize Vector and Ecology Center
