# Supplementary material for: Fipronil and ivermectin treatment of cattle reduced the survival and ovarian development of field-collected Anopheles albimanus in a pilot trial conducted in northern Belize
Source: Malar J. 2019 Aug 29;18:296. doi: 10.1186/s12936-019-2932-6 (PMC6716933; doi:10.1186/s12936-019-2932-6)
Supplement: Supplementary file 1 — Additional file 1. Additional information on comparative toxicities of ivermectin versus permethrin for the STECL laboratory strain of Anopheles albimanus. [file 12936_2019_2932_MOESM1_ESM.docx]

**Additional Information**

**Table S1. Laboratory strains of *Anopheles albimanus* and *A. stephensi* differ in their susceptibility to ivermectin but not to permethrin.**

| *Anopheles* species / strain | Susceptibility to ivermectin * | Susceptibility to permethrin ** | |
| --- | --- | --- | --- |
|  |  | Trial No. | % Mortality (N) |
| *A. albimanus* STECL | 1468 ng/ml | 1 | 100% (24) |
|  |  | 2 | 100% (33) |
|  |  | 3 | 94% (36) |
| *A. stephensi* STE-2 | 7 ng/ml | 1 | 92% (25) |
|  |  | 2 | 95% (22) |
|  |  | 3 | 97% (36) |
|  |  | 4 | 100% (40) |
|  |  | 5 | 100% (53) |
|  |  | 6 | 95% (44) |

* Four-day oral LC-50 [Ref 28].

^**^ Percent mortality after 30 minute exposure to a glass surface treated with permethrin (@ 21.5 mg permethrin per milliliter of ethanol). No mosquito mortality was observed in control groups exposed to glass surfaces treated with ethanol only.
